# Spatiotemporal patterns of spontaneous movement in neonates are significantly linked to risk of autism spectrum disorders at 18 months old

**DOI:** 10.1038/s41598-023-40368-2

**Published:** 2023-08-24

**Authors:** Hirokazu Doi, Akira Furui, Rena Ueda, Koji Shimatani, Midori Yamamoto, Kenichi Sakurai, Chisato Mori, Toshio Tsuji

**Affiliations:** 1 Graduate School of Biomedical Sciences, Nagasaki University, 1-12-4 Sakamoto, Nagasaki, Nagasaki 852-8523 Japan; 2 School of Science and Engineering, Kokushikan University, 4-28-1 Setagaya, Setagaya-ku, Tokyo, 154-8515 Japan; 3 Department of Information and Management Systems Engineering, Nagaoka University of Technology, 1603-1 Kamitomioka, Nagaoka, Niigata, 940-2188 Japan; 4 Graduate School of Advanced Science and Engineering, Hiroshima University, 1-4-1 Kagamiyama, Higashi-hiroshima, Hiroshima, 739-8527 Japan; 5 Faculty of Health and Welfare, Prefectural University of Hiroshima, 1-1, Gakuen-machi, Mihara, Hiroshima, 734-8558 Japan; 6 Department of Sustainable Health Science, Center for Preventive Medical Sciences, Chiba University, 1-33 Yayoi-cho, Inage-ku, Chiba, 263-8522 Japan; 7 Department of Nutrition and Metabolic Medicine, Center for Preventive Medical Sciences, Chiba University, 1-33 Yayoi-cho, Inage-ku, Chiba, 263-8522 Japan; 8 Department of Bioenvironmental Medicine, Graduate School of Medicine, Chiba University, 1-8-1 Inohana, Chuo-ku, Chiba, 260-8670 Japan

**Keywords:** Autism spectrum disorders, Data mining, Image processing, Statistical methods, Biomedical engineering

## Abstract

Infants make spontaneous movements from the prenatal period. Several studies indicate that an atypical pattern of body motion during infancy could be utilized as an early biomarker of autism spectrum disorders (ASD). However, to date, little is known about whether the body motion pattern in neonates is associated with ASD risk. The present study sought to clarify this point by examining, in a longitudinal design, the link between features of spontaneous movement at about two days after birth and ASD risk evaluated using the Modified Checklist for Autism in Toddlers by their caregivers at 18 months old. The body movement features were quantified by a recently developed markerless system of infant body motion analysis. Logistic regression analysis revealed that ASD risk at 18 months old is associated with the pattern of spontaneous movement at the neonatal stage. Further, logistic regression based on body movement features during sleep shows better performance in classifying high- and low-risk infants than during the awake state. These findings raise the possibility that early signs of ASD risk may emerge at a developmental stage far earlier than previously thought.

## Introduction

Infants spontaneously make body movements without external stimulation from 9–10 weeks after gestation^1,2^. Subcortical centers of motor control such as central pattern generator in brainstem and spinal cord supposedly innervate multiple muscles in concert to generate discernible body motions^3^, and recent studies further indicated that cortical as well as subcortical structures contribute to the emergence of spontaneous movements^4^. Many repertoires of body movement are being observable as early as during the foetal period^2^. However, neural regions responsible for spontaneous movement are still immature and body movement pattern shows prominent changes both in quality and quantity during the first year of life^3,4^.

Among early motor repertoire^5,6^, researchers have paid special attention to a constellation of whole body movements called general movement (GM)^3,4,7,8^. From foetal period onward, infant starts to show “writhing movement”, temporally-coordinated and complex pattern of entire body movement that sometimes creates an impression of “fluency and elegance”^4,7^. Around after three to five months of life, writhing movement gradually disappears, and gets replaced by “fidgety movement” characterized by tiny movements of the neck and limbs in all directions with varying intensity^4^.

Abnormality in neural development can be detected non-invasively by GM assessment (GMA). As an illustrative success, Prechtl and colleagues have reported in a series of studies that large proportion of preterm infants with cerebral palsy show abnormal patterns of GM, such as monotonous and rigid movements and lack of movement varieties^4,7^. GM during infancy is reported to be associated with long-term development of cognitive functions into the school year^9,10^. Several studies have made an attempt to see linkage between GM pattern and psychiatric conditions^11,12^, but the number of such studies is limited so far.

According to DSM-5, autism spectrum disorder (ASD) is a neurodevelopmental condition whose diagnostic criteria includes persistent deficits in social communication and social interaction, and restricted, repetitive patterns of behavior, and interests^13^. Currently, a formal diagnosis of ASD is being made at around three years after birth. However, diagnosis can be delayed due to multiple factors such as comorbidities, and large-scale studies report that the median age of ASD diagnosis is around 4.5 to 5.5 years old^14,15^.

Diagnosis can be made as early as two years of age^16^ when a clinical diagnosis is assisted by well-tested instruments such as Pre-linguistic Autism Diagnostic Observation Schedule (ADOS)^17,18^. In addition, a growing number of studies show that infants later diagnosed as ASD exhibit early signs well before they reach three years old^19–22^. A pioneering study by Osterling et al. that analyzed 1-year old birthday videos of ASD children revealed a lower frequency of fixating on other’s faces in ASD children compared to typically developing ones^23^. At this point, there is no definitive treatment for ASD, but studies have pointed out that early intervention to children at risk of ASD leads to better prognosis and social adaptation^24–27^. Thus, the development of methods to assess early signs of ASD risk is of primary importance in improving the well-beings of children with ASD and their caregivers.

A number of studies have revealed an atypical pattern of motoric control in ASD children. These atypicalities include lateral asymmetry in posture and movement^28,29^, hypotonia^19^, and spastic limb movement^24^. In prospective studies, Landa and her colleagues^30,31^ reported that children with ASD show atypical motor control in both gross and fine movements before 24 months old. Teitelbaum et al. proposed a framework for the early diagnosis of ASD by qualitative assessment of body movement at around five months old^32^. Other studies have also suggested the utility of an atypical pattern of body movement as potential biomarker for early screening of ASD risk^33–37^. However, these studies focused on infants after four months old, and it remains unclear whether an assessment of body movement pattern at the neonatal stage is useful in early screening of children at risk for ASD.

**Figure 1:**
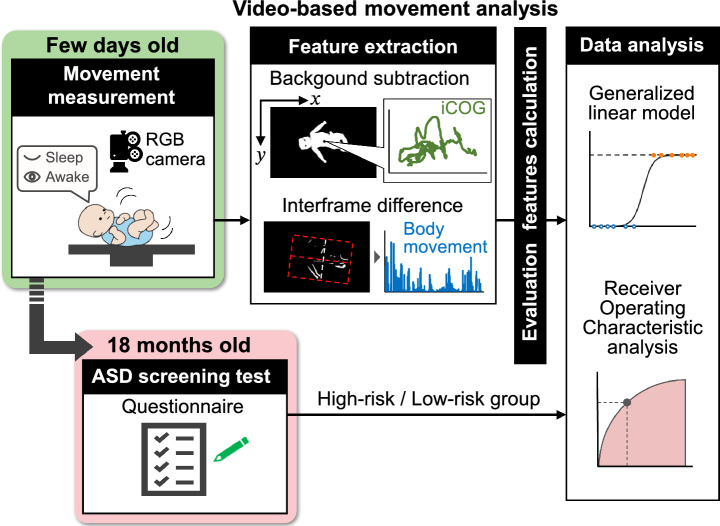
Schematic depiction of the design of present study. The movement of a newborn infant is measured with a camera in the first few days after birth, and evaluation features are calculated through video analysis. At this time, the state of the newborn is distinguished into sleep state and awake state according to the level of arousal. In the video analysis, changes in the image center of gravity (iCOG) are calculated by background subtraction processing, and body movements are calculated by interframe difference processing, obtaining a total of 26 features from them. The relationship between the obtained features and the ASD screening test at 18 months of age is analyzed.

The present study sought to examine whether spontaneous movement in neonates contains information predictive of later emergence of ASD risk. To achieve this end, we quantified characteristics of spontaneous movements in neonates by our markerless system of GM analysis^38^. In a longitudinal design, ASD risk of these infants was evaluated by their caregivers at 18 months old. The main focus of the analysis was to see if ASD risk at 18 months old is associated with spontaneous movement characteristics at the neonatal stage. The overall framework of the present study is schematically described in Fig. 1. Recently, an increasing number of studies have reported the successful application of a markerless system to the analysis of GM (see Silva et al.^39^, for a recent review). Many of these have utilized automatic pose estimation and classification of motion patterns by neural networks^40–43^. Instead, we opted to use feature extraction by computer vision^37,38^ and logistic regression classifier in the present study to develop a classification model with high explainability.

In the present study, patterns of spontaneous movement linked to the later emergence of ASD risk were searched for during sleep and awake state separately. Of particular relevance to the present study, Denisova and Zhao revealed the potential of head movement during sleep as biomarker of ASD risk that emerges during the 1–2 months of life^44^. Furthermore, previous studies on the spontaneous movement of newborns revealed that the pattern of motor control differs depending on arousal state in human and model animals^45,46^.

## Methods

### Participants

75 mother-infant pairs participated in the present study. The cohort included 48 male and 27 female infants. Mothers and fathers were 32.6 ± 4.7 and 34.1 ± 5.8 years old, respectively. Birth weight was 3056.1 ± 323.8 g and gestational age was 39.2 ± 1.2 weeks. Apgar scores were 8.77 ± 0.42 (ranging from 8 to 9) at 1 min and 9.74 ± 0.47 (ranging from 8 to 10) at 5 min. Fifty-five infants were delivered normally, six by vacuum extraction, and nine by cesarean section (information was missing for the remaining five infants). No congenital neurological defects were identified in all infants. Background information such as age and socioeconomic status was collected by a self-administered questionnaire at the time of first enrollment.

The study had been carried out from February 2014 to October 2016 in accordance with the declaration of Helsinki and its protocol had been approved by the institutional ethical committee of Hiroshima University (Approval No. E-1150-2) and Chiba University (Approval No. 451). Written informed consent was obtained from all participants. In the case of infants, informed consent was obtained from a parent and/or legal guardian.

### Procedure

This study was carried out as a part of a long-term longitudinal study^47^. The mothers enrolled in the study during pregnancy. The video-recording of spontaneous body movement took place at a gynecological clinic in north-western area of Chiba prefecture, Japan, during 1–4 days after the delivery. The averaged days between delivery and video-recording was $2.13 \pm 0.66$.

Newborn was taken out from the newborn nursery, and laid in a supine position wearing a diaper on a bed in an adjacent room. Caregiver was absent from the room in which the video recording took place. A video camera was fixed by a special equipment on a transparent ceiling of the bed so that the camera was directed downward towards the neonate. The camera’s location was adjusted to capture the newborn’s entire body and facial expressions. Care was taken to remove any unintended influences on the infant’s state, such as lighting condition or attention-grabbing objects (e.g., moving toys) within the visual field of the newborns^48^. The frame rate was 30 fps, and the resolution was set to $W \times H = 720 \times 480$ pixels. Neonate was left alone under this setting and his/her body movement was recorded for about 18–22 mins. Nurses were instructed to make a minimum level of interventions to and observations of the neonate and refrain from making loud noises nearby the bed.

When the infant reached 18 months, a set of questionnaires was sent out to the mothers, and they were asked to answer the Japanese translation of M-CHAT^49–51^. The Japanese version of M-CHAT is reported to show good test-retest reliability of $r = 0.99$ and external validity^50^. We did not make a follow-up telephone interview that is recommended in ASD risk screening by M-CHAT^49–51^. Mothers submitted the questionnaires after answering all the items.

### Analysis

#### Extraction of body movement features

Quantitative features characterizing the pattern of spontaneous movement were extracted from the video-recordings. From the original video-recordings, temporal segments were deleted during which the infant cried/fussed or apparently slept, or nurses made external stimulation to the infant. A video-segment with deep sleep was defined as a segment during which the eyes were closed, and the infants showed no discernible movement. The entire video-recordings were visually inspected by R.U. and A.F., and final decisions were made by R.U. regarding which video-segments should be submitted for further analysis. The features extracted from the video-recordings are tabulated in Table 1. The exact definition and extraction procedure of each feature are described in more details in our previous studies^37,38^ and are briefly summarized in Supplementary Materials. Because the video recording took place in a hospital room, there was ambient background noise, such as voices and operational sounds, throughout the recording session. The infants seemed to have habituated to such continuous noise. We decided to retain these time-segments because discarding them results in too small number of time-segments available for feature extraction. Abrupt onset of high-level noise, such as noises accompanying door opening or closing, might influence the pattern of an infant’s body movement^48^. We deleted video-segments with high-level noise with abrupt onset.

**Table 1:** Description of the evaluation indices.

Category	Index	Description
a. Movements magnitude	${^{(A_5)}I_1}$, ${^{(A_6)}I_1}$	Movements frequency
	${^{(A_5)}I_2}$, ${^{(A_6)}I_2}$	Movements strength
	${^{(A_5)}I_3}$, ${^{(A_6)}I_3}$	Movements count
b. Movements balance	${^{(A_5,\; A_6)}I_4}$	Ratio of index ${^{(A_5)}I_1}$ in upper body and index ${^{(A_6)}I_1}$ in lower body
	${^{(A_5,\; A_6)}I_5}$	Ratio of index ${^{(A_5)}I_2}$ in the upper body and index ${^{(A_6)}I_2}$ in the lower body
	${^{(A_5,\; A_6)}I_6}$	Symmetry in upper body and lower body
c. Movements rhythm	${^{(A_5)}I_7}$, ${^{(A_6)}I_7}$	Rhythms of the motor alteration $^{(A_k)}M$
	${^{(A_5)}I_8}$, ${^{(A_6)}I_8}$	Standard deviations of indices ${^{(A_5)}I_{7}}$ and ${^{(A_6)}I_7}$
	$^{(A_9)}I_{9_{x}}$, ${^{(A_9)}I_{9_{y}}}$	Rhythms of the iCOG velocities $(G^{\textrm{v}}_{x},~G^{\textrm{v}}_{y})$
	$^{(A_9)}I_{10_{x}}$, $^{(A_9)}I_{10_{y}}$	Standard deviations of index $^{(A_9)}I_{9_{x}}$ and ${^{(A_9)}I_{9_{y}}}$
	${^{(A_9)}I_{11_{x}}}$, $^{(A_9)}I_{11_{y}}$	Rhythms of the iCOG fluctuations $(G^{\textrm{d}}_{x},~G^{\textrm{d}}_{y})$
	$^{(A_9)}I_{12_{x}}$, $^{(A_9)}I_{12_{y}}$	Standard deviations of index ${^{(A_9)}I_{11_{x}}}$ and $^{(A_9)}I_{11_{y}}$
d. iCOG movements	$^{(A_9)}I_{13_{x}}$, $^{(A_9)}I_{13_{y}}$	Variations in the iCOG velocities
	$^{(A_9)}I_{14_{x}}$, $^{(A_9)}I_{14_{y}}$	Standard deviations of the iCOG fluctuations
	$^{(A_9)}I_{15}$	Closed area in the outermost circumference of the iCOG fluctuations

iCOG: Image center of gravity.

The superscript $A_5$ in each index means that the index is computed for the upper body. Similarly, $A_6$ and $A_9$ stand for the lower body and the entire body, respectively.

#### Grouping of infants and group comparison

Based on the scores of M-CHAT administered at 18 months old, infants were classified into high and low ASD risk groups. High risk group included infants who either scored (i) three or more out of 23 items, or, (ii) one or more in the ten critical items^50,51^. These criteria were adopted from the cut-off values used in a large-scale study using the Japanese version of M-CHAT (first stage screening; Kamio et al.^52^). The remaining infants were classified into low risk group. Data from infants with small body movement, ${}^{(A_9)}I_1 \le 5\%$ (proportion of segments with above-threshold movement frequency is equal to or below 5%), were discarded, resulting in 57 low risk and 16 high risk infants. We set the criteria of ${}^{(A_9)}I_1 \le 5\%$ based on the results of visual inspection of the videos; Infants with ${}^{(A_9)}I_1 \le 5\%$ showed virtually no discernible body movements. Supplementary Figure S2 shows the histogram of $^{(A_9)}I_1$ for all infants.

High risk group included 12 male and 4 female infants, while low risk group included 35 males and 22 females. There was no significant difference in the distribution of sex between high and low risk group ($p = 0.387$; Fisher’s exact test). No significant group difference was found between low and high risk infants by Brunner-Munzel test in gestational age, weight at birth, days after birth at video-recording, and weight at the point of video recording, as summarized in Table 2. Weight data at the point of recording was missing for one infant. Thus, group comparison of this variable was conducted for the remaining 56 infants.

**Table 2:** The means and standard deviations (SD) of physiological indices for each group.

Physiological index	Mean ± SD		*p*-value
	Low risk ($n=57$)	High risk ($n=16$)	
Gestational age (day)	277.8 ± 8.1	276.4 ± 9.7	0.58
Weight at birth (g)	3,059.6 ± 337.2	3034.7 ± 290.5	0.93
Days after birth at recording	2.1 ± 0.7	2.2 ± 0.6	0.67
Weight at recording (g)	2949.8 ± 324.4$^\dagger$	2930.1 ± 294.1	0.97

$\dagger$: This index was calculated over 56 subjects because of missing data.

The *p*-values indicate the statistical test results based on the Brunner-Munzel test.

#### ASD risk group classification by logistic regression

We developed a classifier to predict whether an infant belongs to high or low ASD risk group based on the 26 body movement features. These features were selected because our previous study^37^ succeeded in predicting ASD risk classification in 4-month-olds by entering these features into machine learning. Since several of the previous studies found a differential pattern of motor control depending on arousal state^44–46^, we created two separate logistic regression classifiers based on body movement features during time-segments of sleep and awake states. Arousal state was determined by eye closure as the criterion. Specifically, we defined periods during which the eyes were opened as “awake state”, and the remaining periods as “sleep state”. Arousal state during the video segment was determined in a frame-by-frame manner by R.U. Dataset of sleep included recordings from 40 infants (33 low risk infants with a male:female ratio of 22:11 and 7 high risk infants with a male:female ratio of 6:1) whereas the dataset of awake state from 55 infants (41 low risk infants with a male:female ratio of 25:16 and 14 high risk infants with a male:female ratio of 11:3).

Logistic regression was conducted to search for body movement features that differentiate high and low risk infants. The probability *q* with which an infant belongs to high-risk group according to our criteria was modeled by the generalized linear model described below. Body movement features were standardized before being entered into the model. Sex was also included in the model as a binary dummy variable.

1 $$\begin{aligned} g(q)&= \beta _0 + \beta _1 {}^{(A_5)}I_1 + \beta _2 {}^{(A_6)}I_1 + \cdots + \beta _{26} {}^{(A_9)}I_{15} + \beta _{27} \textrm{Sex} \end{aligned}$$

2 $$\begin{aligned} g(q)&= \log \frac{q}{1-q} \end{aligned}$$

Intercept, $\beta _0$ and regression coefficients, $\beta _1$–$\beta _{27}$, were estimated by maximum likelihood estimation so as to maximize joint probability of all the observations in the dataset. Among the 26 features extracted from video recording, a set of body movement features was searched for by a forward-stepwise variable selection procedure to reduce redundancy and retain only informative features. Features to be retained were determined based on the Akaike information criterion (AIC)^53^. More specifically, a set of features that gives the smallest AIC was retained in the model. The classification performance of the resultant model was evaluated by the area under the curve (AUC) in the receiver operation characteristic (ROC) analysis. Classification threshold was defined so that the *F*-measure is maximized. Sensitivity and specificity were calculated at this threshold.

## Results

The model with the smallest AIC is described below for sleep and awake state, respectively. AIC of the resultant model was 31.642 for sleep and 55.629 for awake state. Statistical results for the final model in each state are summarized in Table 3.

**Table 3:** Results of statistical evaluation of resultant model for sleep (upper table) and awake state (lower table). ${}^{(A_5)}I_8$ : Standard deviation of rhythm of the motor alteration in upper body, ${}^{(A_6)}I_7$: Rhythm of the motor alteration in lower body, ${}^{(A_{5,6})}I_4$ : Ratio of movement frequency in upper body and in lower body, ${}^{(A_9)}I_{12x}$ : Standard deviation of rhythm of the image center of gravity fluctuation in vertical axis.

Variable	$\beta$-estimate	SE	*Z*-value	*p*-value
Sleep state				
Intercept	-2.8984	0.9611	-3.016	0.00256 **
${}^{(A_5 )}I_8$	1.7276	0.6890	2.507	0.01217 *
${}^{(A_6 )}I_7$	-2.7631	1.3727	-2.013	0.04412 *
Awake state				
Intercept	-1.2496	0.3656	-3.41	0.000649 ***
${}^{(A_{5,6})}I_4$	1.2332	0.5196	2.373	0.017634 *
${}^{(A_9)} I_{12x}$	0.5201	0.3530	1.473	0.140696

SE: standard error, *$p <0.05$, **$p<0.01$, ***$p<0.001$.

**Figure 2:**
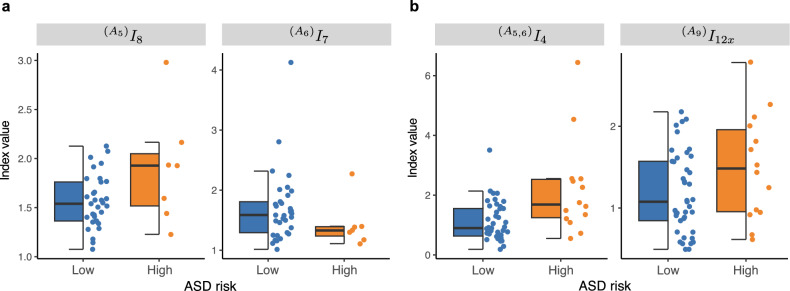
Boxplots of selected evaluation index. (**a**) Sleep state. (**b**) Awake state. ${}^{(A_5)}I_8$ : Standard deviation of rhythm of the motor alteration in upper body, ${}^{(A_6)}I_7$: Rhythm of the motor alteration in lower body, ${}^{(A_{5,6})}I_4$ : Ratio of movement frequency in upper body and in lower body, ${}^{(A_9)}I_{12x}$ : Standard deviation of rhythm of the image center of gravity fluctuation in vertical axis.

As can be seen in the table, higher ASD risk was linked to a larger standard deviation of rhythm of the motor alteration in the upper body, $^{(A_5)} I_8$, and smaller rhythms of the motor alteration in the lower limb, $^{(A_6)} I_7$ during sleep. At the same time, higher ASD risk was associated with a larger ratio of movement frequency in the upper body and in the lower body, $^{(A_{5,6})}I_4$, and a higher standard deviation of rhythm of the image center of gravity fluctuation in the vertical axis, $^{(A_9)} I_{12_x}$ during awake state. Boxplots of retained body movement features in high- and low-risk groups are shown for sleep and awake state in Fig. 2. In any models, sex was not retained as a significant predictor.

**Figure 3:**
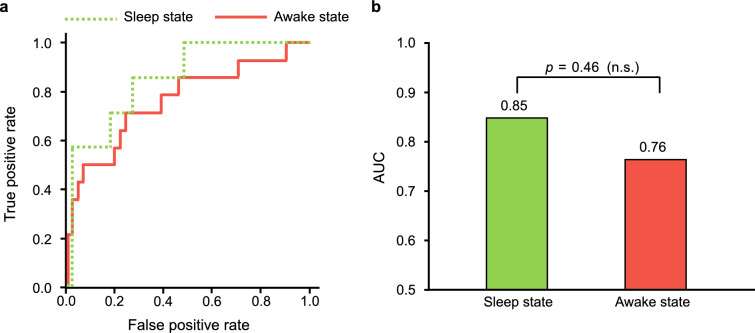
Results of ROC analysis. (**a**) ROC curve for each state. (**b**) AUC value for each state. The statistical test result for AUC using the DeLong’s test are shown.

The ROCs and AUCs of the resultant models are shown in Fig. 3. The AUCs were compared between sleep and awake state, but the Delong's test failed to reveal a significant difference at a significance threshold of 0.05.

**Figure 4:**
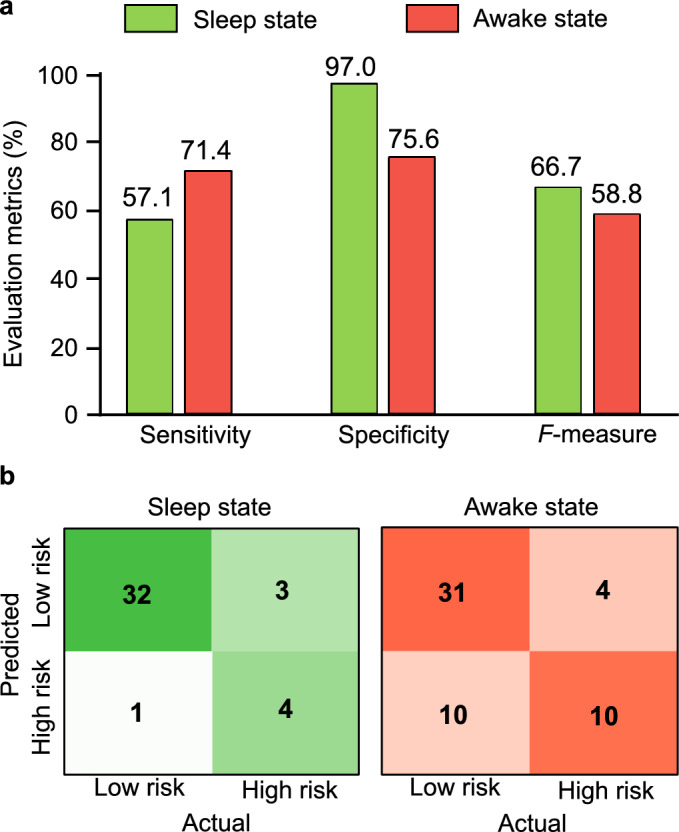
Results of performance indicators. (**a**) Evaluation metrics for each group. The numbers on top of each bar are the exact values of metrics of classification performance evaluation, i.e. sensitivity, specificity and *F*-measure. (**b**) Confusion matrix for each group.

Performance indicators, i.e. sensitivity, specificity and *F*-measure, and confusion matrices at the cut-off value are summarized in Fig. 4. AUC and *F*-measure indicate that classification based on body movement features during sleep show numerically superior performance as evaluated by *F*-measure. This is largely due to higher specificity in sleep than awake state whereas sensitivity was higher in awake state. Classification performance based on the data during sleep was even superior to classification based on all the data combining datasets from both sleep and awake states.

## Discussion

The present study investigated whether the pattern of spontaneous movement in neonates contains information predictive of ASD risk at 18 months old. To achieve this goal, the body movement pattern of neonates was quantified by our markerless movement analysis system^37,38^, and the association was examined between body motion characteristics at the neonatal stage and ASD risk evaluated by the Japanese version of M-CHAT at 18-months old.

During awake state, neonates with high ASD risk showed relatively larger motion frequency in the upper body compared to the lower body, and a larger standard deviation in center of gravity movement along the vertical axis. The former observation is consonant with our previous finding that four-month-olds with high ASD risk show reduced strength of lower limb motions^37^. These observations raise the possibility that ASD risk might be screened by focusing on muscle tone and motion in the lower limb during the first four months after delivery.

Large standard deviation around central frequency in neonates with high ASD risk was observed along the vertical axis in the present study, whereas a similar observation was obtained in 4-month-olds along the medial-lateral axis^37^. The cause for this difference remains elusive at this point. Three-to-four months after delivery is a transitory period during which the dominant pattern of spontaneous motion changes in qualitative as well as quantitative terms, as illustrated in the replacement of writhing by fidgety GM^4^. The body axis along which a lack of rhythmicity in high ASD risk infants is observed differs between neonates and four 4-month-olds probably due to such timeline in motor development.

Performance of logistic regression classifiers was numerically higher in sleep state than awake state. This observation resonates with the findings by Denisova and Zhao that a more frequent occurrence of noisy head movement in 1–2 month-olds with high than low ASD risk infants was prominent during sleep compared to awake state^44^. Classification performance in sleep was even higher than that of the logistic regression classifier trained by all the data combining sleep and awake state, which indicates the importance of considering arousal state in ASD risk prediction based on body movement features. One reason for the numerical difference in classification performance between sleep and awake state is the unbalanced size of the training dataset. At the same time, it is equally possible that body movement makes a differential contribution to neural development depending on arousal state, which claim gains some support from the observation that a different set of features was retained in the logistic regression model in sleep from awake state.

During sleep state, neonates with high ASD risk exhibited larger standard deviation around the central frequency in the upper body and lower central frequency in the lower body. As stated above, larger standard deviation in the distribution of frequency power indicates a lack of a prominent rhythmic component in body motion. This feature can be used as a marker of ASD risk irrespective of the infant’s arousal state. People with ASD are less adept at making rhythmic motion in sync with external stimulation (for a review, see Tordjman et al.^54^) which this ability is of primary importance in socio-emotional and sensori-motor development during early infancy^55–57^. It is conceivable that a lack of rhythmicity in body movement at the neonatal stage is an early sign of atypical rhythmic movement generation in ASD people.

Smaller central frequency in lower limb motion during sleep was associated with a higher ASD risk. Recent studies have suggested that the occurrence of a twitch, a sporadic and jerky movement, of faces and limbs plays primary roles in the fine-tuning of the sensorimotor map in rodents and humans alike^45,46^. Interestingly, a twitch is observed predominantly during sleep at the neonatal stage^45,46^. Based on these, one potential explanation for our observation is that neonates with high ASD risk make a smaller number of high-frequency twitches during sleep.

Neural function underlying the generation of body motion differs between sleep and awake states. For example, afferent input is inhibited during awake state and functional coupling between motor command and proprioceptive information is enhanced during sleep^45,46^. The widely-practiced procedure of GMA deals with the qualitative evaluation of spontaneous movement during wakefulness. Taking these into consideration, it is important to note that our findings alone do not tell whether there is an inherent linkage between body movement during sleep and the development of higher-order cognitive functions. Previous studies on motor function during sleep reported a potential contribution of body motion during sleep to the fine-tuning of somatotopic representation^56–59^. However, it remains to be seen whether the development of other domains of neural functions, such as socio-emotional functions, are causally linked to motor-related neural activity during sleep in infants.

There are several limitations that qualify the interpretation of the present findings. First and foremost, several previous studies question the reliability of M-CHAT as a screening tool for ASD risk^60–62^; Children with a score of 3 to 7 are generally categorized as a group with medium risk for ASD. Worse, we did not carry out a follow-up telephone interview^49^, which must have inflated the false positive rate that infants were classified into high risk group in the present study. Inada et al.^50^ recommended criteria slightly more lenient compared to the original one^63^. For the purpose of lowering the false positive rate, we chose to adopt a more stringent criteria than Inada et al.^50^ following the procedure of the first-stage screening in Kamio *et al*^52^. However, the proportion of high-risk infant was 17.5% (7/40) in sleep state and 25.4% (14/55) in awake state dataset. Both of these values are far larger than the ASD prevalence rate of 1 in 50 reported in previous studies (for example, Xu et al.^64^). Relatedly, the present study included a relatively small number of infants, and thus, high risk infants in the present study probably do not meet the diagnostic criteria of ASD during their toddlerhood, especially because we did not specifically recruit children with a familial risk of ASD. Thus, to validate our main claim that signs of ASD risk emerge at the neonatal stage, it is required in the future study to include infants with high ASD risk who have a genetic liability for ASD, and check whether the infant receives a diagnosis of ASD in a longitudinal design. It is also indispensable to evaluate a participant’s symptoms using an established and more reliable assessment tool such as ADOS.

Second, it is unclear what physiological condition the “sleep state” in the present study corresponds to. In the present study, we classified the neonate’s arousal state based on whether their eyes are opened or not^65^. But with such qualitative criteria, we cannot say with confidence whether sleep as per definition in our study corresponds to sleep or incidental closing of the eyes during awake state. Lastly, the recording environment was not strictly controlled. Since video-recording took place in a hospital, we could not entirely exclude background noises and casual interventions to the infants by hospital staffs, such as looking into the baby bed, during recording. Though video-segments with external stimulation were carefully discarded from the analysis, it is possible that overlooked stimulation from the environment influenced neonates’ arousal state and movement pattern. To address the last two points, it is desirable to record an infant’s body movement under a controlled laboratory environment simultaneously with scalp EEG data for arousal state classification.

Fourth, we deleted data from infants with small or no body movement, which leaves open the possibility that discarded data included infants with the lowest frequency of limb motion. We decided to discard these data because reliable quantification of body movement features was not feasible for these cases. Given the discussion above that reduced frequency of lower limb motion might be an early biomarker of ASD, it is conceivable that we failed to include the most severe case of ASD-related motor atypicality.

Fifth, the sample size of the present study was relatively small. This small sample size might explain the lack of significant contribution of sex in the prediction of ASD risk. Many previous studies found a sex difference in symptoms and behavioral phenotypes of ASD^66–68^. Relatedly, a structural brain imaging study^69^ found notable sex differences in motor-related neural regions. Considering these, it is possible that we could detect the effect of sex in a study with a larger sample size.

## Conclusion

The present study has shown that characteristics of spontaneous movement at the neonatal stage reflects ASD risk evaluated by M-CHAT at 18 months old, which raises the possibility that early signs of ASD emerge at the neonatal stage, far earlier than previously thought. Predictability of ASD risk was higher when classification was made based on body movement features during sleep than awake state. At the same time, the present study suffers from many limitations. Most importantly, the proportion of high-risk infant was much higher than the reported prevalence rate of ASD, which indicates that high-risk group in the present study included many false positives. Thus, the present findings alone do not tell us whether features extracted from spontaneous movement could be utilized as an early biomarker of ASD.

Investigation into the association between early motor development and ASD is still at a nascent stage. Several studies indicate an association between GM and a later diagnosis of ASD (see Einspieler et al.^36^ for a review). However, definitive conclusions cannot be drawn from these studies, as the sample size in many of them is quite small. The introduction of an automated system greatly reduces the cost of GMA. Although preliminary, the present findings support the prospect of large-scale research on the association between early motor development and ASD, assisted by semi-automatic assessment of an infant’s spontaneous movement.

### Supplementary Information

Supplementary Information.

## Data Availability

The datasets used and/or analysed during the current study available from the corresponding author on reasonable request.
