# Supplementary material for: Spatiotemporal patterns of spontaneous movement in neonates are significantly linked to risk of autism spectrum disorders at 18 months old
Source: Sci Rep. 2023 Aug 24;13:13869. doi: 10.1038/s41598-023-40368-2 (PMC10449803; doi:10.1038/s41598-023-40368-2)
Supplement: Supplementary file 1 — Supplementary Information. [file 41598_2023_40368_MOESM1_ESM.pdf]

Supplementary Materials for

Spatiotemporal patterns of spontaneous movement in neonates are significantly linked to risk of autism spectrum disorders at 18 months old

Hirokazu Doi[1,2,3], Akira Furui[4], Rena Ueda[4], Koji Shimatani[5], Midori Yamamoto[6], Kenichi Sakurai[7], Chisato Mori[6,8], and Toshio Tsuji[4,*]

[1]Graduate School of Biomedical Sciences, Nagasaki University, 1-12-4 Sakamoto, Nagasaki, Nagasaki 852-8523, Japan.
[2]School of Science and Engineering, Kokushikan University, 4-28-1 Setagaya, Setagaya-ku, Tokyo 154-8515, Japan.
[3]Department of Information and Management Systems Engineering, Nagaoka University of Technology, 1603-1 Kamitomioka, Nagaoka, Nigata 940-2188, Japan.
[4]Graduate School of Advanced Science and Engineering, Hiroshima University, 1-4-1 Kagamiyama, Higashi-hiroshima, Hiroshima 739-8527, Japan.
[5]Faculty of Health and Welfare, Prefectural University of Hiroshima, 1-1, Gakuen-machi, Mihara, Hiroshima 734-8558, Japan.
[6]Department of Sustainable Health Science, Center for Preventive Medical Sciences, Chiba University, 1-33 Yayoi-cho, Inage-ku, Chiba 263-8522, Japan.
[7]Department of Nutrition and Metabolic Medicine, Center for Preventive Medical Sciences, Chiba University, 1-33 Yayoi-cho, Inage-ku, Chiba 263-8522, Japan.
[8]Department of Bioenvironmental Medicine, Graduate School of Medicine, Chiba University, 1-8-1 Inohana, Chiba, Chiba 260-8670, Japan
* tsuji-c@bsys.hiroshima-u.ac.jp

The PDF file includes:

Supplementary Texts
Supplementary Figure S1–S3

Supplementary Texts:
Extraction of body movement features

S ($1 \leq s \leq S$) video segments eligible for analysis were extracted from the video of each infant. The flow of image processing of each infant's video is schematically shown in Supplementary Figure S1. First, we extracted the infant's body from each frame using U^2-Net [1], and converted each frame into a binary image with pixels in background being 0 (black) and those within body area being 1 (white). The binary body area image in the l-th frame ($l = 1, 2, \ldots, L_s$) within each video segment is hereinafter referred to as $f_l(x, y)$ ($x = 1, 2, \ldots, W; y = 1, 2, \ldots, H$), where L_s denotes the number of frames in s-th video segment, and (x, y) is a coordinate within a frame, with x and y representing location along longitudinal and horizontal body axes, respectively.

Second, body movement was detected by computing interframe difference image $f'_l(x, y)$.

$$f'_l(x, y) = \eta(|b_l(x, y) - b_{l-1}(x, y)|) \tag{1}$$

$$\eta(d) = \begin{cases} 1 & (d \geq d_{\mathrm{th}}) \\ 0 & (d < d_{\mathrm{th}}), \end{cases} \tag{2}$$

where $f'_1(x, y) = 0$, $b_l(x, y)$ is a masked image of l-th frame created by masking l-th frame by $f_l(x, y)$, and $\eta(\cdot)$ is a function to create a binary image of body movement by binarizing $f'_l(x, y)$ with threshold $d_{\mathrm{th}} = 80$.

From the body area image, the body posture ${}^{(A_k)}P_l$, which reflects the body size, was obtained using the following equation.

$${}^{(A_k)}P_l = \sum_{(x,y) \in A_k} f_l(x, y). \tag{3}$$

The body movement ${}^{(A_k)}M_l$, which reflects the amount of body motion relative to body size, was obtained from the interframe difference images as follows:

$${}^{(A_k)}M_l = \frac{1}{{}^{(A_9)}P^{\mathrm{avg}}} \sum_{(x,y) \in A_k} f'_l(x, y). \tag{4}$$

Here, A_k ($k = 1, 2, \ldots, 9$) denotes subregion of infant's body. A_1 corresponds to left upper limb region, and so do A_2, A_3, A_4, right upper limb, left lower limb, right lower limb regions, respectively. A_5 ($= A_1 \cup A_2$), A_6 ($= A_3 \cup A_4$), A_7 ($= A_1 \cup A_3$), A_8 ($= A_2 \cup A_4$), A_9 ($= A_5 \cup A_6$) correspond to upper body, lower body, left body, and entire body respectively. ${}^{(A_9)}P^{\mathrm{avg}}$ is the mean of 10 largest ${}^{(A_9)}P_l$ in the first 30 frames in the first video segment of each infant.

Based on the image center of gravity (iCOG) computed based on $f_l(x, y)$, we calculated iCOG velocity ($G^v_{l,x}, G^v_{l,y}$) and iCOG fluctuation ($G^d_{l,x}, G^d_{l,y}$) by

the following formula.

$$(G_{l,x}, G_{l,y}) = \left(\frac{1}{{}^{(A_9)}P_l} \sum_{(x,y)\in A_9} x f_l(x,y), \frac{1}{{}^{(A_9)}P_l} \sum_{(x,y)\in A_9} y f_l(x,y) \right), \tag{5}$$

$$(G^v_{l,x}, G^v_{l,y}) = \left(\frac{F_s(G_{l,x} - G_{l-1,x})}{\sqrt{{}^{(A_9)}P^{\text{avg}}}}, \frac{F_s(G_{l,y} - G_{l-1,y})}{\sqrt{{}^{(A_9)}P^{\text{avg}}}} \right), \tag{6}$$

$$(G^d_{l,x}, G^d_{l,y}) = \left(\frac{G_{l,x} - G^{\text{avg}}_x}{\sqrt{{}^{(A_9)}P^{\text{avg}}}}, \frac{G_{l,y} - G^{\text{avg}}_y}{\sqrt{{}^{(A_9)}P^{\text{avg}}}} \right), \tag{7}$$

where $(G^v_{1,x}, G^v_{1,y}) = (0,0)$, F_s is the frame rate, and $(G^{\text{avg}}_x, G^{\text{avg}}_y)$ denotes the mean of $(G_{l,x}, G_{l,y})$ in the first 30 frames in the first video segment. Finally, iCOG velocity and iCOG fluctuation were smoothed by second-order Butterworth low-pass filter with cutoff frequency of 5 Hz for iCOG velocity and 10 Hz for iCOG fluctuation.

The total length of video segments differed across participants. The length of video segments to be used for the computation of body movement features was equalized across participants by the procedure described below.

1. ${}^{(A_9)}M_k$ from S video segments were concatenated.
2. Moving windows with a length of 1,800 frames (1 min) and a stride of 1 frame were applied to the concatenated ${}^{(A_9)}M_l$. The movement frequency in entire body area, ${}^{(A_9)}I_1$ (see below), within each window was calculated with a threshold $M_{\text{th}} = 0.01$.
3. Body movement features were computed in the window within which ${}^{(A_9)}I_1$ was maximum.

Twenty-six body movement features were calculated according to the definitions described in Doi *et al.* [2] These features are broadly grouped into four categories, i.e., movement magnitude, movement balance, movement rhythm, and iCOG movement. Definitions of these features are briefly summarized below. Each feature was calculated for the entire S' segments included in the time period under analysis. In the analysis, the parameters M_{th} and $f_{\max}$ were set to 0.005 and 15 Hz respectively.

Movement Magnitude

${}^{(A_k)}I_1$: Movement frequency

This feature is the proportion of frames with body movement above threshold in body area A_k using the following equations:

$${}^{(A_k)}I_1 = \frac{100}{L} \sum_{s=1}^{S'} \sum_{l=1}^{L_s} {}^{(A_k)}\kappa_l, \tag{8}$$

$${}^{(A_k)}\kappa_l = \begin{cases} 1 & ({}^{(A_k)}M_l \geq M_{\text{th}}) \\ 0 & ({}^{(A_k)}M_l < M_{\text{th}}), \end{cases} \tag{9}$$

where L denotes the total number of frames in the first to S'th video segment, and M_{th} is the threshold to judge whether body movement occurred in each frame.

${}^{(A_k)}I_2$: Movement strength

This feature is defined as the averaged strength of body movement across frames with body movement in body area A_k using the following equations:

$$ {}^{(A_k)}I_2 = \frac{1}{L''} \sum_{s=1}^{S'} \sum_{l=1}^{L_s} {}^{(A_k)}\nu_l, \tag{10} $$

$$ {}^{(A_k)}\nu_l = \begin{cases} {}^{(A_k)}M_l & ({}^{(A_k)}M_l \geq M_{\mathrm{th}}) \\ 0 & ({}^{(A_k)}M_l < M_{\mathrm{th}}), \end{cases} \tag{11} $$

where L'' denotes the number of frames with ${}^{(A_k)}M_l \geq M_{\mathrm{th}}$.

${}^{(A_k)}I_3$: Movement count

This feature is defined as the count of discrete body movement occurrences across S' video segments in body area A_k:

$$ {}^{(A_k)}I_3 = \frac{1}{L} \sum_{s=1}^{S} {}^{(A_k)}Q_s, \tag{12} $$

where ${}^{(A_k)}Q_s$ denotes the count of discrete body movement occurrences in the s-th video segment. ${}^{(A_k)}Q_s$ is incremented by one every time ${}^{(A_k)}Q_s$ becomes lower than M_{th} after ${}^{(A_k)}M_l$ gets larger than or equal to M_{th}. ${}^{(A_k)}Q_s$ is incremented by one also when ${}^{(A_k)}M_l$ is larger than or equal to M_{th} at the end of video segment.

Movement Balance

${}^{(A_{k_1},A_{k_2})}I_4$: Ratio between ${}^{(A_{k_1})}I_1$ and ${}^{(A_{k_2})}I_1$

This feature is defined as the ratio of movement frequencies between body areas A_{k_1} and A_{k_2} $(k_1 = 1, 2, \ldots, 8; k_2 = 1, 2, \ldots, 8; k_1 \neq k_2)$:

$$ {}^{(A_{k_1},\,A_{k_2})}I_4 = \frac{{}^{(A_{k_1})}I_1}{{}^{(A_{k_2})}I_1}. \tag{13} $$

Note that if ${}^{(A_{k_2})}I_1 = 0$, then ${}^{(A_{k_1},A_{k_2})}I_4 = 0$.

${}^{(A_{k_1},A_{k_2})}I_5$: Ratio between ${}^{(A_{k_1})}I_2$ and ${}^{(A_{k_2})}I_2$

This feature is defined as the ratio of movement strength between body areas A_{k_1} and A_{k_2} $(k_1 = 1, 2, \ldots, 8; k_2 = 1, 2, \ldots, 8; k_1 \neq k_2)$:

$$ {}^{(A_{k_1},\,A_{k_2})}I_5 = \frac{{}^{(A_{k_1})}I_2}{{}^{(A_{k_2})}I_2}. \tag{14} $$

Note that if ${}^{(A_{k_2})}I_2 = 0$, then ${}^{(A_{k_1},A_{k_2})}I_5 = 0$.

${}^{(A_{k_1},A_{k_2})}I_6$: Symmetry in ${}^{(A_{k_1})}M_l$ and ${}^{(A_{k_2})}M_l$

This feature is defined as the correlation coefficient $\rho(A_{k_1}, A_{k_2})$ between ${}^{(A_k)}M_l$ in body areas A_{k_1} and A_{k_2} ($k_1 = 1, 2, \dots, 8; k_2 = 1, 2, \dots, 8; k_1 \neq k_2$). In the s-th segment, the correlation coefficient is calculated within a window of width $L_c = 300$ frames using the following formula:

$$\rho(A_{k_1}, A_{k_2}) = \frac{\sum_{l=1}^{L_c} \left({}^{(A_{k_1})}M_l - {}^{(A_{k_1})}M^{\text{avg}}\right)\left({}^{(A_{k_2})}M_l - {}^{(A_{k_2})}M^{\text{avg}}\right)}{\sqrt{\sum_{l=1}^{L_c} \left({}^{(A_{k_1})}M_l - {}^{(A_{k_1})}M^{\text{avg}}\right)^2}\sqrt{\sum_{l=1}^{L_c} \left({}^{(A_{k_2})}M_l - {}^{(A_{k_2})}M^{\text{avg}}\right)^2}}, \tag{15}$$

where ${}^{(A_{k_i})}M^{\text{avg}}$ represents he mean value of ${}^{(A_{k_i})}M_l$ within the window. The correlation coefficients were repeatedly calculated within sliding temporal windows. This process was performed for all segments, and finally, all obtained correlation coefficients were averaged to obtain the feature ${}^{(A_{k_1},A_{k_2})}I_6$.

Movement Rhythm

${}^{(A_k)}I_7$: Mean power frequency of ${}^{(A_k)}M_l$

${}^{(A_k)}I_8$: Second moment around mean power frequency ${}^{(A_k)}I_7$

Power spectral density distribution $P(f)$ of ${}^{(A_k)}M_l$ was first computed by Fourier transformation in moving widows with the length $L_f = 128$ frames and stride of 1 frame. Then, $P^{\text{avg}}(f)$ was calculated as the mean of $P(f)$ across all the windows:

$$F_{\text{MPF}} = \frac{\sum_{f=0}^{f_{\max}} f P^{\text{avg}}(f)}{\sum_{f=0}^{f_{\max}} P^{\text{avg}}(f)}, \tag{16}$$

$$D_{\text{MPF}} = \sqrt{\frac{\sum_{f=0}^{f_{\max}} P^{\text{avg}}(f)(f - F_{\text{MLP}})^2}{\sum_{f=0}^{f_{\max}} P^{\text{avg}}(f)}}, \tag{17}$$

where $f_{\max} = 15$ Hz represent upper limit of frequency. The calculated F_{MPF} and D_{MPF} are defined as indices ${}^{(A_k)}I_7$ and ${}^{(A_k)}I_8$, respectively.

${}^{(A_9)}I_{9x}$, ${}^{(A_9)}I_{9y}$: Rhythms of the iCOG velocities (G_x^v, G_y^v)

${}^{(A_9)}I_{10x}$, ${}^{(A_9)}I_{10y}$: Standard deviations of ${}^{(A_9)}I_{9x}$ and ${}^{(A_9)}I_{9y}$

By a similar procedure to $\left({}^{(A_k)}I_7, {}^{(A_k)}I_8\right)$, mean power frequency F_{MPF} of (G_x^v, G_y^v) was calculated as $\left({}^{(A_9)}I_{9x}, {}^{(A_9)}I_{9y}\right)$ and its standard deviation D_{MPF} as $\left({}^{(A_9)}I_{10x}, {}^{(A_9)}I_{10y}\right)$.

${}^{(A_9)}I_{11x}$, ${}^{(A_9)}I_{11y}$: **Rhythms of the iCOG velocities** (G^d_x, G^d_y)

${}^{(A_9)}I_{12x}$, ${}^{(A_9)}I_{12y}$: **Standard deviations of** ${}^{(A_9)}I_{11x}$ **and** ${}^{(A_9)}I_{11y}$

By a similar procedure to $\left({}^{(A_k)}I_7, {}^{(A_k)}I_8\right)$, mean power frequency F_{MPF} of (G^d_x, G^d_y) was calculated as $\left({}^{(A_9)}I_{11x}, {}^{(A_9)}I_{11y}\right)$ and its standard deviation D_{MPF} as $\left({}^{(A_9)}I_{12x}, {}^{(A_9)}I_{12y}\right)$.

iCOG movement

${}^{(A_9)}I_{13x}$, ${}^{(A_9)}I_{13y}$: **Variation in the iCOG velocities** (G^v_x, G^v_y)

This feature is defined as the mean of the absolute values of iCOG velocities from the 1st to S'-th video segments:

$$\left({}^{(A_7)}I_{13x}, {}^{(A_7)}I_{13y}\right) = \left(\frac{1}{L}\sum_{s=1}^{S'}\sum_{l=1}^{L_s}\left|G^{\mathrm{v}}_{l,x}\right|, \ \frac{1}{L}\sum_{s=1}^{S'}\sum_{l=1}^{L_s}\left|G^{\mathrm{v}}_{l,y}\right|\right). \tag{18}$$

${}^{(A_9)}I_{14x}$, ${}^{(A_9)}I_{14y}$: **Standard deviations of the iCOG fluctuations** (G^d_x, G^d_y)

This feature is defined as the standard deviation of iCOG fluctuation computed within sliding temporal windows of the length $L_g = 300$ frames and stride of 1 frame by the following formula:

$$\sigma_j = \sqrt{\frac{1}{L_{\mathrm{g}}}\sum_{l=1}^{L_{\mathrm{g}}}\left(G^{\mathrm{d}}_{l,j} - \overline{G}^{\mathrm{d}}_j\right)^2}, \tag{19}$$

where $j \in \{x, y\}$, and $\overline{G}^{\mathrm{d}}_j$ represents the average value of $G^{\mathrm{d}}_{l,j}$ within the window. The σ_j was calculated in all video sub-segments, in the same way; then, I_{14_i} is calculated by averaging all σ_j.

${}^{(A_9)}I_{15}$: **Closed area in the outermost circumference of the iCOG fluctuations** (G^d_x, G^d_y)

Closed areas of the outmost circumference of the trajectory of iCOG fluctuations (G^d_x, G^d_y) was calculated within moving windows of the length $L_g = 300$ frames and stride of 1 frame following the procedure by Kim *et al.* [3]. In each window, the points on the outermost circumference of the trajectory were arranged in a clockwise order. These points are denoted as $(G^d_{b,x}, G^d_{b,y})$ ($b = 1, 2, \ldots, B$; B denotes the total number of points on the outermost circumference). Then, the area within the outermost circumference was calculated by the following formula.

$$\frac{1}{2}\sum_{b=1}^{B}\left|G^{\mathrm{d}}_{b,x}G^{\mathrm{d}}_{b+1,y} - G^{\mathrm{d}}_{b,y}G^{\mathrm{d}}_{b+1,x}\right|. \tag{20}$$

Note that if $b = B$, then $b + 1 = 1$. Feature ${}^{(A_9)}I_{15}$ was computed as the mean area across all the windows.

References

[1] Qin, X., Zhang, Z., Huang, C., Dehghan, M., Zaiane, O. R., Jagersand, M., U^2-Net: Going deeper with nested U-structure for salient object detection, *Pattern Recogn.* **106**, 107404 (2020).

[2] Doi, H., Iijima, N., Furui, A., Soh, Z., Yonei, R., Shinohara, K., Iriguchi, M., Shimatani, K., Tsuji, T., Prediction of autistic tendencies at 18 months of age via markerless video analysis of spontaneous body movements in 4-month-old infants, *Sci. Rep.* **12**, 18045 (2022).

[3] Kim, G., Ferdjallah, M., Harris, G. F., Fast computational analysis of sway area using center of pressure data in normal children and children with cerebral palsy, *Am. J. Biomed. Sci.* **1**, 364–372 (2009).

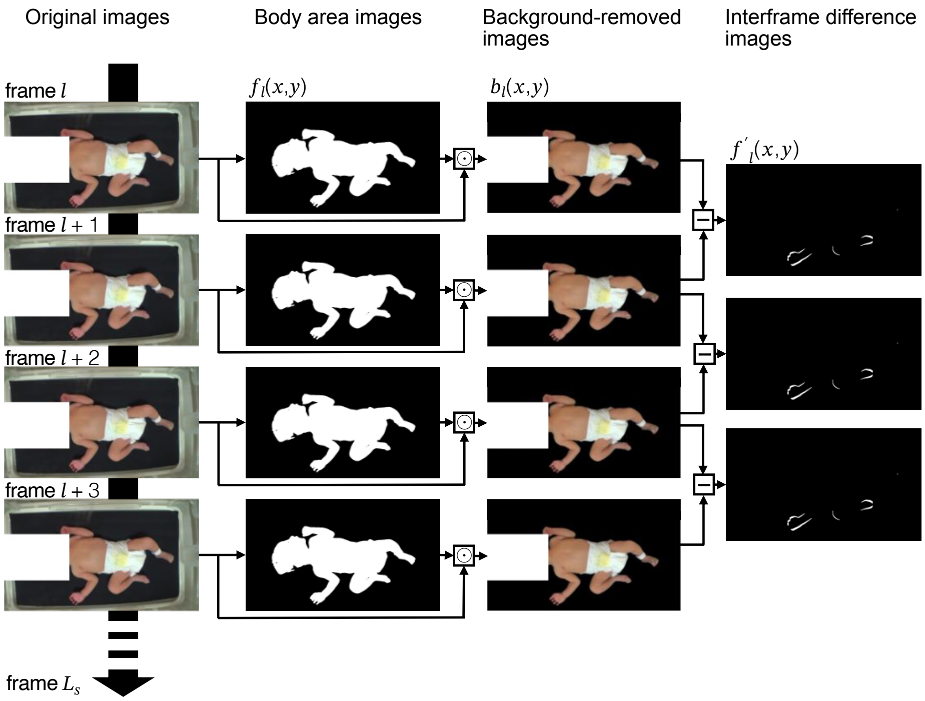

Supplementary Figure S1: Procedure of image processing

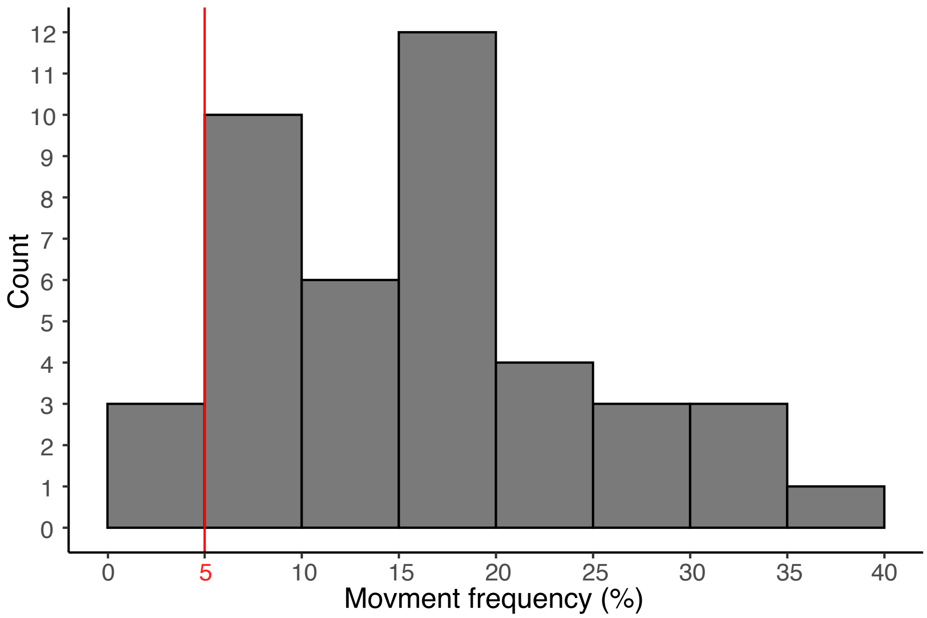

Supplementary Figure S2: Histogram of whole-body movement frequency, denoted by ${}^{(A_9)}I_1$, for all infants. Most infants show clear movement for 10–20% of the entire video's duration. To exclude data with almost no movement, we included only infants whose movement frequency exceeded 5% in our analysis.

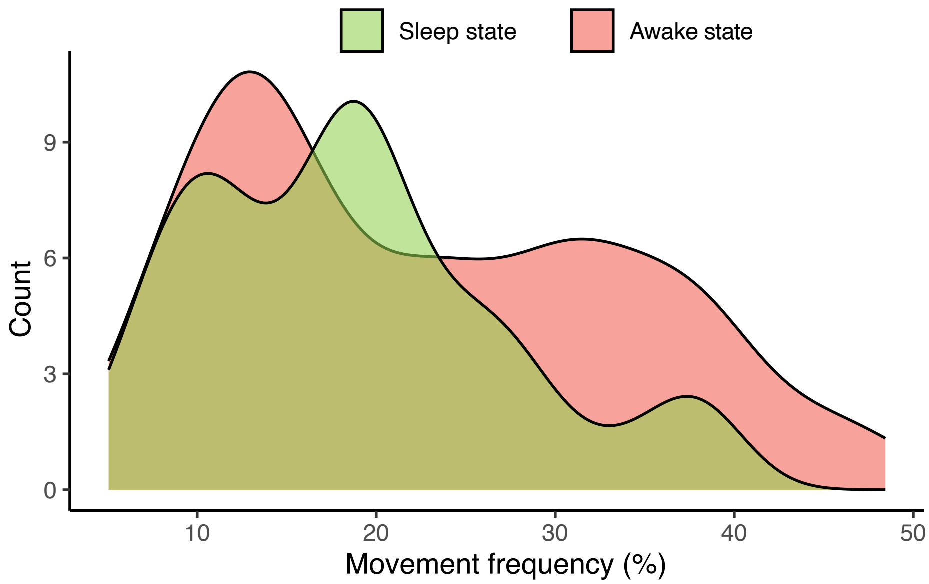

Supplementary Figure S3: Density histograms of the whole-body movement frequency, denoted by ${}^{(A_9)}I_1$, for both the sleep state and awake state data. The movement frequency in the sleep state was mainly concentrated in the lower 20%, whereas that in the awake state peaked at around 13% and between 30% and 40%. This suggests that the quantitative (or qualitative) characteristics of movements differ depending on the infant's state.
